# Luteolin loaded PEGylated cerosomes: a novel treatment for MRSA skin infections

**DOI:** 10.1186/s12866-025-03873-0

**Published:** 2025-03-31

**Authors:** Sally A. Mohamed, Walaa A. Eraqi, Paris E. Georghiou, Mohamed Y. Zakaria

**Affiliations:** 1 Microbiology and Immunology Department, Faculty of Pharmacy, Cairo University, Cairo, 11562 Egypt; 2 Department of Chemistry, Memorial University of Newfoundland, St. John’s, Newfoundland and Labrador, A1B 3X7 Canada; 3 Department of Pharmaceutics and Industrial Pharmacy, Faculty of Pharmacy, King Salman International University, Ras Sudr, 46612 South Sinai Egypt; 4 Department of Pharmaceutics and Industrial Pharmacy, Faculty of Pharmacy, Port Said University, Port Said, 42526 Egypt

**Keywords:** MRSA, Luteolin, PEGylation, Cerosomes, Biofilm, Skin infection model

## Abstract

**Background:**

Methicillin-resistant *Staphylococcus aureus* (MRSA) is a major cause of skin and soft tissue infections which, due to the spread of antimicrobial resistance, have become increasingly serious. Bacterial skin infection affects the barrier function of skin causing depletion of the ceramide content in the stratum corneum (SC) of the epidermis. In the study reported herein, luteolin (LUT) a naturally-occurring flavonoid was incorporated in PEGylated cerosomes (PCs) to boost its antibacterial action as a topical application. The opimal formulation of the surface-modified lipidic vesicles was chosen with the aid of a 2^3^ full factorial design. The effectiveness of the optimal LUT formulation which was developed was evaluated using several MRSA strains both in vitro and in vivo studies.

**Results:**

A 2^3^ full factorial design was employed for the preparation of the optimum PC formulation, designated herein as F5. A comparative in vitro release study revealed the superiority of F5 over a LUT suspension in solubilizing and releasing after 24 h, a higher percentage 78.1 ± 1.8% of luteolin compared with only 18.3 ± 2.1% for the luteolin suspension. When tested against MRSA strains, F5 showed antimicrobial activity that was higher than that of the luteolin suspension, having a MIC value of 187.5 µg/mL versus 1500 µg/mL. In addition to having enhanced anti-virulence activity than the luteolin suspension in terms of antibiofilm formation (with % inhibition ranging from 45 to 99% with the tested strains at 0.5 × and 0.25 × MICs, where the luteolin suspension only had a range from 1 to 45%), enhanced anti-pigment production, and anti-α-hemolysin activity were also observed. Moreover, F5 affected the cell wall integrity as confirmed by transmission electron microscopy (TEM). Scanning electron microscopy (SEM) verified the effect of F5 on bacterial biofilm formation, showing reduction of cellular adhesion and disruption of biofilm, factors which greatly contribute to bacterial pathogenesis and antibiotic resistance. When compared to the negative control and the luteolin suspension groups, the F5 formulation also resulted in reducing the bacterial load in the murine skin infection model.

**Conclusions:**

F5 PEGylated cerosomes are potential new potent defense agents against MRSA infections, demonstrating promising therapeutic capabilities.

## Background

*Staphylococcus aureus* (*S. aureus*) is a common member of the skin's natural microbiota which asymptomatically colonizes approximately 30% of the human population [1]. It is one of the ESKAPE pathogens, a group that also includes *Enterococcus faecium, Klebsiella pneumoniae, Acinetobacter baumannii, Pseudomonas aeruginosa*, and *Enterobacter* species [2]. *S. aureus* produces various virulence factors, including toxins such as hemolysins and leukocidins, immune-evasive surface components such as the capsule and protein A, as well as enzymes such as hyaluronidase that facilitate tissue invasion [3]. This adaptable human pathogen has the capacity to induce a variety of diseases, ranging from minor skin and soft tissue infections to more serious infections such as osteomyelitis, pneumonia and life-threatening sepsis in both community and healthcare settings worldwide [4–6].

Methicillin-resistant *Staphylococcus aureus* (MRSA) is a subgroup of *S. aureus* that displays resistance to a broad range of beta-lactam antibiotics, penicillins and most cephalosporins, in addition to many other antibiotics such as erythromycin, clindamycin, gentamycin and ciprofloxacin [7]. Methicillin resistance is mediated by the *mecA* gene which is acquired through the horizontal transfer of the staphylococcal cassette chromosome *mec* (SCC*mec*), which encodes PBP2a, enabling Staphylococci to evade β-lactam antibiotics [8].

MRSA is a major cause of nosocomial and community-acquired infections worldwide that can lead to higher mortality rates compared to that caused by methicillin-sensitive *S. aureus* [9, 10]. Individuals with MRSA infections are 64% more prone to die from their infection than those with drug-sensitive infections [11]. Treatment of MRSA infections presents a substantial challenge, as only a limited selection of antibiotics, including vancomycin, teicoplanin, linezolid, and tigecycline remain effective [12, 13]. Notably, several newly discovered strains of MRSA exhibit resistance even to vancomycin and teicoplanin, rendering these last-resort antibiotics less effective [14, 15]. Consequently, the U.S. Centers for Disease Control and Prevention (CDC) has classified MRSA as a significant threat, emphasizing the critical need to find effective additional or newer treatments [16].

The COVID-19 pandemic has further exacerbated antimicrobial resistance (AMR). Approximately 70% of COVID-19 infected patients who were, motivated by the fear of co-infections during the pandemic, received antimicrobial agents [17, 18]. The unnecessary use of antibiotics however has further amplified AMR concerns. CDC reports indicated a 14% rise in MRSA infections in 2019–2020, with considerable economic costs and a concerning halt in the decline of MRSA cases after 2017 [19]. The World Health Organization (WHO) featured antibiotic resistance as one of the top 10 threats facing humanity [20]. The 2021 Global Health Security (GHS) Index Country Profile reveals that the more affluent countries had much higher AMR indicator scores (e.g. 69.8 for Canada) compared with the average Global AMR of 38.9 and for example Egypt (16.7) in 2019 and 2021 [21].

Without implementing an effective plan to address AMR, it is expected that the annual death toll from infections caused by multidrug resistant (MDR) pathogens could reach 10 million by the year 2050, exceeding the mortality rate for cancers and all other causes of death [10]. This requires urgent actions to be taken to achieve the Sustainable Development Goals [22] and there is an urgent demand to develop new antimicrobial agents that enable a decrease in the use of antibiotics. Without urgent action, humans could be heading toward a post-antibiotic era, in which common infections and minor injuries can once again become highly lethal [23].

These concerns have also led to a growing interest in exploring natural products as a promising source for combating antibiotic resistance. Natural products, derived from various sources such as plants, marine organisms, and microorganisms, have demonstrated a remarkable potential to provide novel solutions against antibiotic-resistant pathogens [24, 25]. The study reported herein focuses on employing Luteolin (LUT), a naturally-occurring flavonoid which has anti-inflammatory, anticancer, and antioxidant properties [26–28].

Skin infections including MRSA infections are chiefly managed by either the administration of antimicrobial agents via systemic oral administration and/or using a topical application. Although oral administration of antimicrobial agents is widely known to be more effective, this route unfortunately commonly predisposes affected individuals also to an increased risk of drug-drug interactions, toxic side effects [29]. Hence, the use of topical applications is of great interest to avoid those risks.

Due to their ultra-elastic characteristics, lipidic vesicular systems have been proposed as successful carriers for the administration of many bioactives through the dermal route. Cerosomes (Cs) which are ceramide-enriched vesicles that are prepared using phospholipids along with various surfactants for stabilization issues, are being proposed to be used in many pharmaceutical areas since this nano-carrier is characterized by having good accommodation and dissolution of ceramide which acts as a vital component in this system. Ceramides are a class of sphingolipid, which maintain the integrity and protective role in the outermost layer of the skin i.e. the stratum corneum (SC). It has been reported that the addition of up to 0.05% w/w of ceramide in pharmaceutical formulations augments the ability of the skin in renewing its natural protective layer and in restricting skin moisture escape. Furthermore, it has also been reported that in many skin diseases such as psoriasis, topical skin infections and eczema cause drastic changes in the levels of ceramide in the skin which can affect its condition [30]. Thus the incorporation of ceramide in topical preparations has proven to be very beneficial, especially in treating many skin diseases and for the restoration of the skin’s barrier function [31].

For this study, polyethylene glycolated (PEGylated) cerosomes (PCs) were developed and and evaluated as new vesicular carriers for LUT as a potential effective topical treatment of MRSA skin infections. Additionally, Brijs® (PEGylated surfactants) were utilized in the formulation as the edge activator (EA) which impart an extra elasticity to the vesicles to enable the deep penetration of the drug through the skin layers. Additionally, PEGylation of the prepared systems may extend their deposition and residence time in the skin and thus, preserving the target drug concentration over a long period of time in the skin and finally, enhancing the efficacy of topically administered drugs [32].

## Materials and methods

### Materials

Luteoline (LUT) and L-a-phosphotidylcholine from egg yolk were purchased from Sigma Aldrich Chemical Co. (St. Louis, MO, USA). Ceramide IIIB was kindly supplied as a gift from Evonic Co. (Germany). Brij52 (polyoxyethylene (2) cetyl ether) and BrijO20 were purchased by BASF Co. (New Jersy, NY, USA). All solvents were obtained from El-Nasr Pharmaceutical Chemicals Co. (Cairo, Egypt).

### Methods

#### Formulation of LUT-loaded PCs

The Thin Film Hydration technique (TFH) was employed for each of the PCs formulations thar are summarized in Table 1 [33, 34]. A typical procedure employed LUT (20 mg), egg yolk phospholipid (150 mg), Ceramide IIIB (25 mg) and Brij52 (20 mg) which were then dissolved in a 2:1 (V/V) chloroform**:**ethanol mixed solvent (10.0 mL) in a round-bottomed flask. The flask was rotated on a rotatory evaporator for 30 min at 90 rpm, under vacuum for 30 min to ensure the slow and complete evaporation of the organic phase and to allow the formation of a thin film onto the wall of the flask. The thin film was then hydrated with distilled water (10.0 mL) with rotation, at a temperature of 60 °C which is higher than the transition temperature of the lipid phase (Tc) for 1 h. The vesicular dispersion was finally stored overnight at 4 °C for further characterizations.

**Table 1:** 2^3^ full factorial experimental design; Experimental runs, independent variables, and estimated responses of LUT-loaded PCs

**Formula**	**A** Ceramide (mg)	**B** (EA type)	**C** (EA amount) (mg)	**Y1** (EE%)	**Y2** (PS) (nm)	**Y3** (ZP) (mV)	**PDI**
F1	25	Brij 20	20	58 ± 2.1	132 ± 16.3	-25 ± 1.9	0.2 ± 0.08
F2	50	Brij 20	20	69 ± 2.6	190 ± 18.1	-11 ± 1.1	0.3 ± 0.05
F3	25	Brij 20	40	43 ± 1.7	89 ± 5.4	-15 ± 5.2	0.1 ± 0.02
F4	50	Brij 20	40	55 ± 3.1	119 ± 10.4	-5 ± 0.8	0.2 ± 0.02
F5	25	Brij 52	20	81 ± 1.9	228 ± 20.4	-45 ± 2.6	0.2 ± 0.07
F6	50	Brij 52	20	91 ± 2.4	298 ± 19.6	-31 ± 2.4	0.8 ± 0.08
F7	25	Brij 52	40	71 ± 1.2	202 ± 20.5	-36 ± 3.7	0.4 ± 0.09
F8	50	Brij 52	40	82 ± 1.9	255. ± 23.1	-26 ± 3.3	0.3 ± 0.06

#### In vitro analysis and optimization of the formulated LUT-loaded PCs

##### Evaluation of PCs EE%

Precise amounts (1.0 ml) of the prepared formulae dispersion were centrifuged at 15,000 rpm at 4 °C for 1 h in a cooling centrifuge. The concentration of LUT entrapped in the sedimented vesicles was then determined spectrophotometrically after the vesicles were disrupted using methanol (Shimadzu UV1650 Spectrophotometer, Koyoto, Japan), at λ_max_ 350 nm [35]. All measurements were conducted in triplicate.

##### Evaluation of particle size (PS), polydispersity index (PDI) and zeta potential (ZP)

The average PS and PDI of the dispersion formulae after being diluted (1:100) were determined using a Malvern Zetasizer 2000 (Malvern Instruments Ltd., UK) [36, 37]. Moreover, using the same equipment the ZP was determined by assessment of the electrophoretic movement of the particles in the electrical field. All measurements were conducted in triplicate.

##### Selection of the optimal LUT-loaded PCs

The impact of changing the independent variables on two levels on the physical characteristics of prepared PCs was analyzed via a 2^3^ full factorial design. Those investigated independent variables were the amount of ceramide used (A), the EA type (B) and the EA amount (C). The EE%, the PS, the PDI and the ZP values for each vesicle’s physical characterizations were chosen as the dependent variables. The design resulted in construction of eight different formulae considering all possibilities of the independent variables levels (Table 1). The optimization process was performed based on the following criteria: the highest EE% and ZP values (absolute values) in addition to the smallest PS and lowest PDI. The formula with highest desirability value was selected as the optimum formula. The responses of the dependent variables of the assumed optimized formula were then compared with predicted responses in order to affirm the validity of the model [38].

#### In vitro characterization of the optimized LUT-loaded PC

##### Differential Scanning Calorimetry (DSC)

Thermal investigation of 5.0 mg samples, from each sample of LUT, lyophilized plain optimized formula and lyophilized LUT-loaded optimized formula was conducted under an inert nitrogen flow with a DSC instrument (DSC-60, Shimadzu Corp., Kyoto, Japan and standardized with purified indium over a temperature range of 10–400 °C at a scanning rate of 5 °C/min [39].

##### Comparative in vitro release study

Two milliliters of each of the optimized LUT-loaded PC and the LUT suspension were placed in plastic cylindrical tubes. Each tube has a specific permeation area of one of its ends firmly covered with a pre-soaked cellulose membrane. The other end is attached to the rotating shaft of a USP Dissolution apparatus. The suspensions were rotated at 50 rpm for 24 h at 37 °C ± 0.5. The dissolution flask contains 200 ml of PBS pH 7.4 from which 5.0 ml samples were then withdrawn at 1, 2, 4, 6, 8, 12 and 24 h [40]. Finally, the samples were analyzed by UV spectrophotometry at λ_max_ 350 nm. Each experiment was repeated in triplicate.

#### Bacterial strains and culture conditions

Methicillin resistant *S. aureus* (MRSA) USA300, and clinical isolates MS3, MS15, MS16, MS23 obtained from the Department of Microbiology and Immunology culture collection, Faculty of Pharmacy, Cairo University, identified by Gram-stain, catalase production, oxidation/fermentation pattern, coagulase, DNase, gelatinase testing, API Staph-test (Bio-Merieux, Paris, France) and Dry Spot Staphytect Plus kit (Oxoid, Hants, UK). Growing the isolates on ORSAB medium identified the isolates as MRSA, as they showed blue colonies. In addition, the isolates were tested for the presence of *mecA* gene and were subjected to molecular typing by ERIC-PCR [41]. The bacterial strains were cultivated on Mannitol Salt Agar plates (MSA), a selective medium for *S. aureus* (Oxoid, Hants, UK) and were incubated at 37 °C for 24 h. Strains were grown aerobically at 37 °C in tryptic soy broth (TSB) (Oxoid, Hants, UK).

#### Determination of MICs and MBCs of the tested formula against MRSA isolates

MICs were determined according to [42], in accordance with the Clinical and Laboratory Standards Institute (CLSI) guidelines [43], with slight modifications. Briefly, two-fold serially diluted optimized luteolin F5 formula, or luteolin suspension in double-strength Mueller Hinton (MH) broth (Oxoid, Hants, UK), in concentrations starting from 1500 µg/mL to 2.93 µg/mL. To each dilution, 10.0 µL of bacterial suspension with a final concentration of 5 × 10^4^ CFU/mL was added. The bacterial suspension was prepared by transferring 2–3 pure colonies from an overnight culture of the tested strains into sterile physiological saline solution, adjusted to 0.5 McFarland then diluted to reach a bacterial concentration of about 5 × 10^6^ CFU/mL. Double-strength MH broth alone was used as a negative control, and MH broth inoculated with 10.0 µL of bacterial suspension with an inoculum size of 10^6^ CFU/mL was used as a positive control. The 96-well plates were incubated at 37 °C for 18 h. MICs were determined as the lowest concentration showing no bacterial growth and determinations were conducted in triplicate. Aqueous (0.5%) triphenyltetrazolium chloride (TTC) (Oxoid, Hants, UK) solution was used for confirmation of bacterial growth. Ten microliter amounts of freshly prepared TTC aqueous solution (0.5%) was added to the 96-well plates' mixtures and incubated at 37 °C for 30 min. The yellow color of TTC solution is changed by viable bacterial cells to pink or red 1,3,5- triphenylformazan (TPF). For MBC determination, ten microliters of the mixtures of each bacterial suspension and the tested formulas up to the concentration of the MIC, were spotted on MH agar plates and incubated at 37 °C for 24 h. The MBC was determined as the lowest concentration of formula showing no bacterial growth [9]. The test was performed in triplicate.

#### Antivirulence activity of luteolin

##### Biofilm inhibition assay

Biofilm inhibition assays were performed according to Zakaria and coworkers [42]. Briefly, overnight cultures of MRSA strains in TSB were adjusted to OD_600_ = 1 suspensions, diluted in fresh TSB to reach a final count of 10^8^ CFU/mL. Fifty microliters of diluted cultures were put onto 96-well microtiter plates, to which 50 µL of optimized F5 formula were also added, such that the final concentration of the formula would be half of its MIC with the respective strains. After 24 h incubation at 37 °C, the optical density of the overnight cultures was measured (Planktonic) at 600 nm and then the plates were decanted, washed twice with sterile phosphate buffered saline (PBS), and dried. Crystal violet solution (0.4%) was used to stain the adherent cells for 30 min at room temperature. The plates were washed three times and 100 µL of 99% ethanol was finally added to the wells and left for 30 min at room temperature with gentle shaking. The OD absorbance was measured at 595 nm. The test was performed in triplicate. Tryptic soya broth was used as blank, and TSB inoculated with bacteria was used as a positive control. The biofilm formation index (BFI) was calculated as follows:

$$BFI=(ODcv\;Biofilm-ODcv\;Control)/OD\;Planktonic$$

##### Anti-pigment production activity against selected MRSA strains

The effect of the optimized F5 formula on the pigment production of the MRSA strains was qualitatively evaluated according to Zhang et al. [44]. Briefly, overnight cultures of standard strain MRSA USA300 and Clinical Isolates MS3, MS15, MS16 and MS23 in TSB, both untreated and treated with 0.25 × or 0.5 × MIC of the optimized luteolin formula were centrifuged at 10,000 × g for 10 min. The formed pellets were washed twice with PBS and were visually examined for pigment production. The untreated cultures were used as positive controls.

##### Anti- α-hemolysin activity against the selected MRSA strains

The anti-α-hemolysin effect of the optimized F5 formula was determined according to Zhang et al. [44]. Briefly, the overnight cultures of standard strain MRSA USA300 and Clinical Isolates MS3, MS15, MS16 and MS23 in TSB, both untreated and treated with 0.25 × or 0.5 × MIC of optimized F5 formula were pelleted. In 1.5 mL Eppendorf tubes, 25 µL of freshly withdrawn defibrinated rabbit RBCs was added to 875 µL PBS. To the prepared RBCs suspension, 100 µL of supernatant (untreated or treated) was added, gently mixed, and incubated at 37 °C for 15 min, then centrifuged at 10,000 × g for 2 min at 20 °C. The percentage of hemolysis was measured at 543 nm using a microplate reader (Synergy 2, BioTek, WI, USA). PBS-only treated RBCs were used as negative control, while Triton-X 100-treated RBCs were used as a positive control (100% hemolysis). Percentage of hemolysis was calculated according to the following equation:

$$\%\;hemolysis=\left({Sample\;OD}_{543}-{Negative\;control\;OD}_{543}\right)/{Positive\;controlOD}_{543}\times100$$

#### Assessment of potential disruption of F5 to both biofilm formation and cell wall structure of MRSA USA300

##### Use of electron microscopy to visualize the effect of F5 on the biofilm of MRSA USA300

MRSA USA300 standard strain was incubated in TSB broth overnight at 37 °C with shaking at 180 rpm. After incubation, the OD_600_ was adjusted to 0.5. In a six-well plate, a volume of 250 µl of the F5 formula at its minimum inhibitory concentration was added to an equal volume of the adjusted bacterial culture to have a final concentration equal to 0.5 MIC, and the plate was incubated for 24 h at 37 °C. In another set of wells in the plate, 250 µl of the drug-free formulation was incubated with an equal volume of TSB broth as a negative control. After incubation, samples were prepared for scanning electron microscopy (SEM) photography at a resolution of 5.5 nm and magnification power of 15 × 200,000 × on a scanning electron microscope (JEOL model JSM-5200F, 25 kV, Tokyo, Japan) at the Faculty of Agriculture, Cairo University, Egypt [10].

##### Use of transmission microscopy to visualize the effect of F5 on MRSA USA300 cells

Morphological changes in the bacterial cells of MRSA USA300 treated with the F5 formulation were examined using transmission electron microscopy (TEM). An overnight culture of the bacterial isolate in TSB broth was adjusted to OD_600_ of one, then, MIC of the Luteolin F5 formula was added and incubated for 24 h. After incubation, bacterial suspension was centrifuged, washed twice with PBS, and prepared for TEM photography according to a previously published protocol [45]. TEM photography was conducted with a Flash electron microscope, 120 kV (JEOL model JEM-1400 JEOL Ltd., Tokyo, Japan) with a resolution of 0.2 nm and magnification power of 10 × to 1,200,000 × , at the Faculty of Agriculture, Cairo University, Egypt.

#### In-vivo murine skin infection model

##### Ethical statement

The protocol of the study was approved by the Research Ethics Committee at the Faculty of Pharmacy, Cairo University, Egypt (Approval Numbers: MI 3053), following the guidelines for the Care and Use of Laboratory Animals published by the Institute of Laboratory Animal Research (USA).

##### Experimental design

An in-vivo MRSA skin infection model study was conducted according to Elsebaie et al. [46]. Briefly, 6–8 weeks-old female Balb/C mice weighing 25–30 g were blindly distributed into four groups (*n* = 6) which comprised the following: Group I: F5 formulation; Group II: luteolin dispersion; Group III: 2% fusidic acid commercial ointment (positive control) and Group IV: Petroleum jelly (negative control). The animals were kept in cages under well-defined and standardized conditions and were fed with a standard dry food and water. Back hair was shaved with an electric hair clipper one day before infection. The four groups were subcutaneously injected with 100 µL of MRSA USA300 (1–2 X 10^9^ CFU) suspended in sterile pyrogen-free saline. Seventy-two hours post infection, the site of infection was treated with either, petroleum jelly containing formula (5 × its MIC), petroleum jelly containing luteolin suspension (5 × its MIC) or 2% fusidic acid. The treatment was applied topically twice daily for 4 days. Twenty-four hours after the last treatment (wash out), the mice were sacrificed after being euthanized with an overdose of 2,2,2- tribromoethanol in 2-methyl-2-butanol (Sigma, MI, USA) followed by cervical dislocation. Spleens and skin infection samples were aseptically removed using sterile instruments for determination of viable counts. The skin or spleen tissues were homogenized in 1.0 mL of sterile saline using tissue homogenizer. Ten-fold serial dilutions of each sample were then prepared in saline (20 µL of the organism mixed with 180 µL saline) in a 96-well plate. From each dilution, 10 µL was spotted onto MSA plates and incubated overnight at 37 °C. The viable count (CFU/mL) was calculated using the following equation: VC (CFU/mL) = (number of colonies / dilution factor) x (100). All procedures were performed in accordance with AVMA euthanasia guidelines to ensure minimal distress to the animals.

#### Statistical analysis

Data were analyzed and plotted using GraphPad Prism software (version 9.0) (GraphPad Software, Inc., USA). Data were presented as mean ± standard deviation (SD) of three independent biological experiments. Two-way ANOVA test with Tukey’s multiple comparison was used to compare the results of anti- α-hemolysin activity. One-way ANOVA followed by Tukey’s Multiple Comparisons test was used for comparing the effect of F5 and LUT dispersion in the murine animal model. Multiple unpaired t-test was used for analysis of antibiofilm activity results. *p*-values < 0.05 were considered significant.

## Results and discussion

### Factorial design statistical evaluation

Different formulation variables (amount of ceramide) (Factor A), type of EA (Factor B) and amount of EA (Factor C) were selected to be evaluated in our study using the experimental design via Design-Expert Software *version 13.23*. A full factorial design was adopted to study the influence of the formulation variables and preparation and optimization of different LUT-loaded PCs. The design resulted in eight experimental runs considering the different levels of the experimental variables (Table 1). The adequacy of the experimental model to navigate in the design space was affirmed by the appropriate precision value [47]. As shown in Table 2, precision values > 4 could be noticed for all the dependent variables. The difference between the predicted and adjusted R2 should not be > 0.2 in order to indicate a good harmony between the values which also reflects the suitability of the design. An adequate agreement between the predicted R2 values and the adjusted R2 for all the dependent variables could be depicted from Table 2.

**Table 2:** 2^3^ Factorial analysis data of LUT-loaded PCs; predicted, observed responses and deviation percentages of F5

Responses	EE (%)	PS (nm)	ZP (mV)
R^2^	0.9999	0.9996	1
Adjusted R^2^	0.9994	0.9974	1
Predicted R^2^	0.9946	0.976	0.9997
Adequate precision	132.2	61.6	594.9
Significant factors	A, B, C	A, B, C	A, B, C
Observed value of the optimal formula (F5)	80.9	228.3	-44.5
Predicted value of the optimal formula (F5)	80.7	226.9	-44.5
Absolute deviation %	0.25	0.61	0

### Effect of formulation variables on the entrapment efficiency (EE%)

**Fig. 1:**
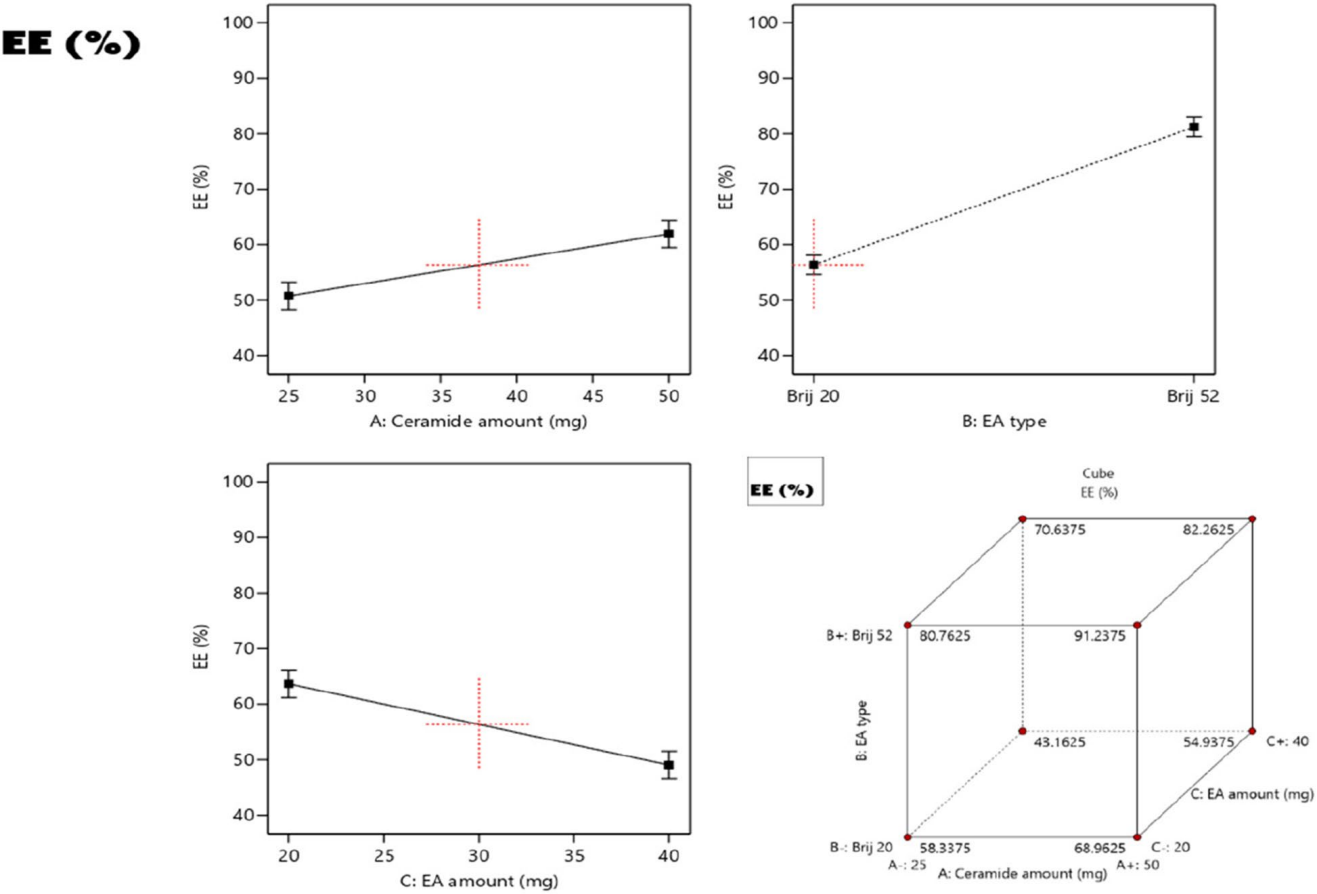
The effect of independent variables on EE% of LUT-loaded PCs

The potential of the prepared vesicular system to serve as a promising nano-carrier for topical delivery of LUT is highly dependent on their ability to encapsulate a reasonable amount of the drug. In this study, the incorporation of the phosphatidylcholine along with the lipophilic ceramide as key ingredients in the formulation guaranteed the entrapment of adequate amounts of the water-insoluble LUT within the fabricated PCs. As demonstrated in Table 1, the values of EE% of the prepared LUT-loaded PCs were in the range of 43 ± 1.7 to 91 ± 2.4%. Moreover, ANOVA statistical analysis showed that all the investigated variables exhibited a significant impact on the EE% (Fig. 1).

The increase from 25 to 50 mg in the amount of ceramide (Factor **A**) in the preparations positively affected the EE% of the vesicles (*p* < 0.0157). Using higher amounts of ceramide in the formulations has been reported to increase the viscosity of the formulation dispersions [34]. An increase in the system viscosity will prohibit the drug diffusion throughout the vesicular membranes, thus higher EE% values were achieved. With regard to EA type (Factor B), the ANOVA results revealed that the EE% values of the formulations prepared using Brij52 were significantly higher compared to those prepared using BrijO20 (*p* = 0.007). This may be related to the difference in the hydrophilic-lypophilic balance (HLB) values of Brij52 (HLB = 5.3) and BrijO20 (HLB = 15.3). EAs having lower HLB values aid in increasing the integrity and entrapment efficiency (EE%) of the vesicles to the lipophilic LUT, thus Brij52 resulted in higher EE% than BrijO20 [48]. Moreover, the differences in the structures of the EAs (i.e. differences in chain length, degree of unsaturation of the alkyl chain, and number of PEG repeating units) can greatly affect the EE%. Brij52 consists of a C16-saturated alkyl chain (derived from cetyl alcohol) with an average of two repeating PEG units, whereas BrijO20 (Brij98) consists of a C18-monounsaturated alkene chain, derived from cis-9-octadecen-1-ol (oleyl alcohol) linked with an average of twenty repeating PEG units. The unsaturation in the alkene chain results in a decrease in the degree of packing of the vesicles, and to the formation of leakier vesicles. Hence the EE% values with BrijO20 were much lower than those with Brij52 [33, 49]. Increasing the amount of EA from 20 to 40 mg on the other hand, resulted in significant decline in EE% values (*p* = 0.0145). This impact may be attributed to the formation of more porous vesicles and the increase in the bilayer fluidity on using higher amounts of surfactants which will result in increased drug leakage rate and a subsequent decrease in EE% values [50].

### Effect of formulation variables on the particle size (PS) and PDI

**Fig. 2:**
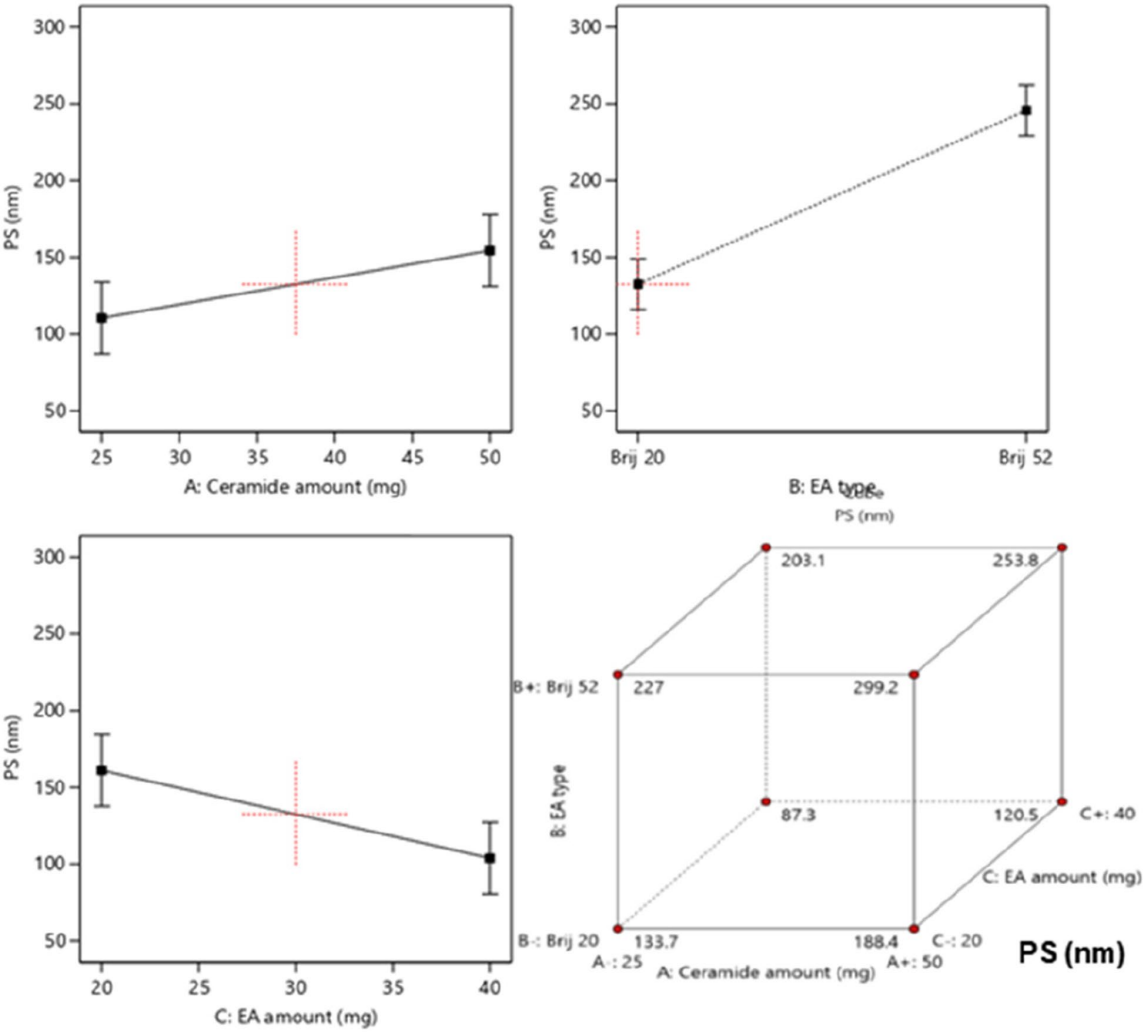
The effect of independent variables on PS of LUT-loaded PCs

The formulation of a stable vesicular system relies highly upon the particle size (PS) of the vesicles and their tendency for fusion and aggregation, thus the production of vesicles of small PS is very crucial in guaranteeing the system stability. Furthermore, the extent of drug permeation across the skin layers and its retention in the layers are highly affected by the particle size of the nanosystem [51].The PS range of the prepared LUT-loaded PCs was 89 ± 5.4 to 298 ± 19.6 nm (Table 1). Moreover, ANOVA statistical analysis declared that all the investigated variables exhibited significant effect on the PS (Fig. 2).

The incorporation of a higher amount of ceramide (**A**) resulted in a significant increase in the PS of the vesicles (*p* < 0.0314). The increase in ceramide amount as previously mentioned results in an increase in the viscosity which also increases the tendency of the vesicles to aggregate; hence larger particles were produced [48]. Furthermore, the structural changes in the membranes of the vesicles induced by the ceramide addition resulted in prominent alterations in the curvature of the membranes and an increase in PS owing to the low ability of ceramide to diffuse throughout the layers of the vesicles [52]. Additionally, significantly larger PS were derived using Brij52 than those prepared using BrijO20 (*p* = 0.0146). This could be due the difference in HLB values of both surfactants. The surfactant with the higher HLB (BrijO20) reduces the surface free energy of the system, thus reducing the PS of the vesicles. Moreover, the difference in PEG units, namely 2 and 20 respectively, in the structures of surfactants Brij52 and BrijO20 are also deemed to have a great effect upon the PS. Increasing the number of repeating PEG units in the surfactant leads to a decrease in the tendency of the vesicles towards aggregation and sedimentation, thus improving the stability of the vesicles and producing vesicles of smaller PS [47]. Finally, ANOVA statistical analysis revealed that increasing the amount of EA (Factor **C**) resulted in a subsequent significant reduction in PS of the vesicles (*p* = 0.036). This could be attributed to the augmentation in the curvature of the vesicles and the suppression in interfacial tension of the system achieved by using higher amounts of EA and thus lowering the PS. It has previously been reported that increasing the amount of EA aids in covering all of the lipids and hence lowering the surface tension. Moreover, using higher amounts of EA permits the accumulation of a larger number of PEG units surrounding the vesicles which prohibits the aggregation of the vesicles and hence enhances steric stability of the particles and reduces the PS [53]. Furthermore, the quality of the fabricated nano-vesicles and the homogeneity of their dispersions could be deduced from their PDI values. Whereas lower PDI values closer to zero denote homogenous dispersion and a narrow PS range, higher PDI values closer to 1 denote the polydispersity and heterogeneity of the system [33]. As shown in Table 1, the PDI values of the formulated PCs were in the range of 0.23 ± 0.06 to 0.78 ± 0.08 which denotes the heterogeneity of some of the formulated PCs. These high PDI values may be attributed to the irregular vesicular shape of cerosomes. ANOVA analysis revealed that all the inspected factors exhibited a non-significant influence on the PDI of the systems, thus PDI was not considered in the optimization process [54].

### Effect of formulation variables on the zeta potential (ZP)

Zeta potential (ZP) determinations are crucial for exploring the total surface charges on the surfaces of nano-vesicles, for anticipating their physical stability [50], and for any interactions which may occur with the physiological membranes in the body [55]. Table 1 shows that the ZP values of all the prepared PCs ranged from -5.0 ± 0.8 to 45 ± 2.6 mV.

**Fig. 3:**
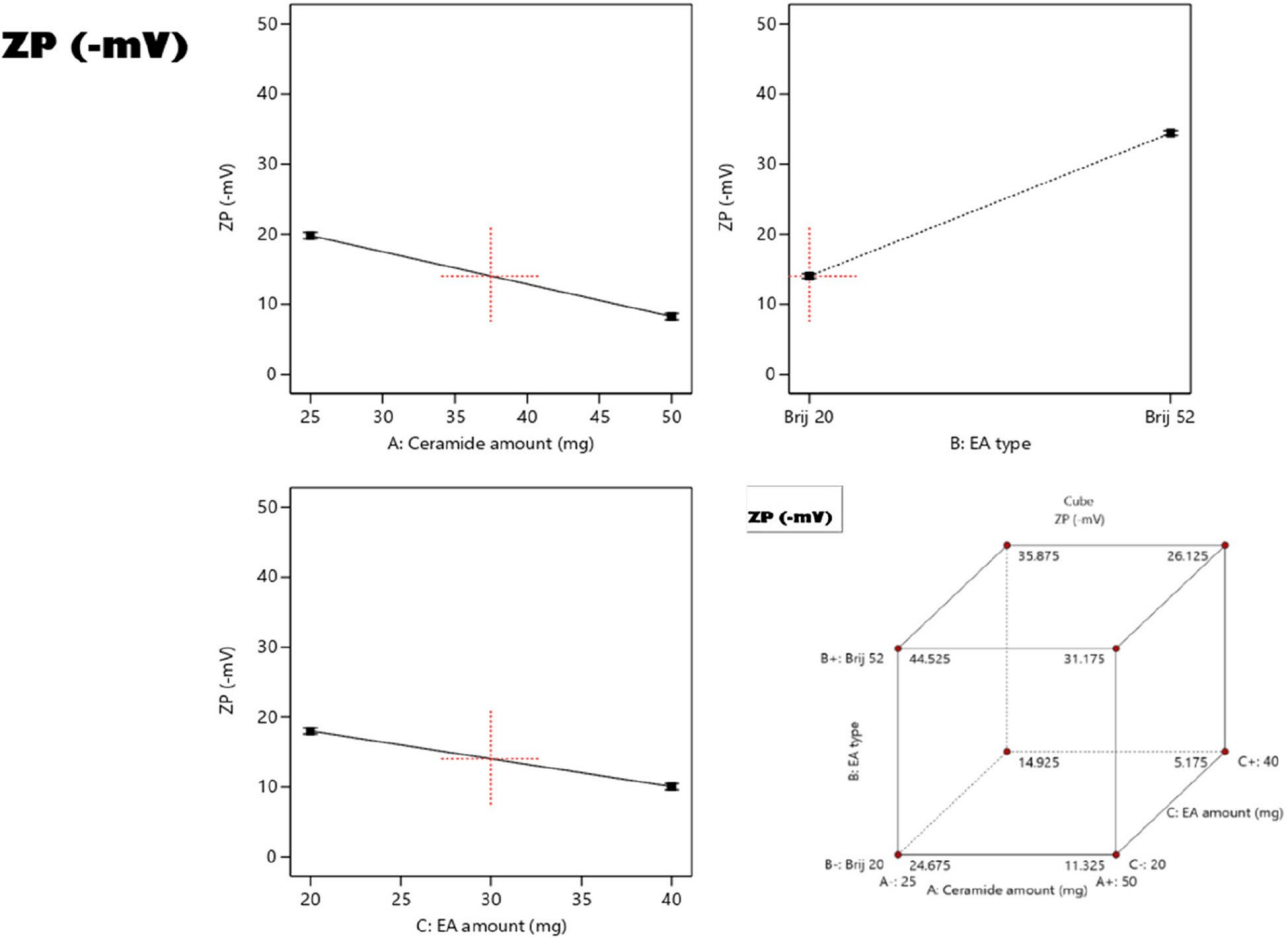
The effect of independent variables on ZP of LUT-loaded PCs

Additionally, ANOVA statistical analysis declared that all the investigated variables exhibited significant effects on the ZP (Fig. 3). Increasing the amount of ceramide (Factor **A**) resulted in a significant decline in the absolute values of ZP (*p* = 0.0028). This could be rationalized based on the accumulation of ceramide on the surface of the formulated vesicles and which in turn owing to ceramide’s amphiphilic property will mask the charges of the prepared PCs and lower their ZP values [52]. On the contrary, PCs formulated using Brij52 exhibited significantly higher absolute values of ZP than those prepared using BrijO20 (*p* = 0.0016). This finding can be attributed to the fact that BrijO20 has a higher number of PEG units than Brij52 which arrange themselves on the outermost layer of the vesicles thus shielding the surface charges of the vesicles, leading to lower ZP values [56]. In addition, increasing the amount of EA (Factor **C**) also resulted in significantly lower absolute values of ZP (*p* = 0.0043), since higher numbers of hydrophilic PEG units will be accumulated at the surface of the vesicles which will consequently shield the negative charges of PCs [57].

### Selection of the optimal LUT-loaded PCs

The analysis of the potential outcomes of the dependent variables that resulted from using different levels of the four independent variables and the determination of the optimum levels of those independent variables were conducted utilizing Design Expert® software. From this analysis, formulation F5 was predicted to be the optimal formula with the highest desirability value (0.643). The optimum formula was compounded using 25 mg of ceramide, and 20 mg Brij52 having a high LUT EE% of 80.9 ± 1.9, nanosized vesicles of 228.3 ± 20.4 nm, and the highest ZP value of -44.3 ± 2.6 mV (absolute value). Additionally, the actual responses of the dependent variables of F5 were in good agreement with the predicted responses as shown in Table 2, thus demonstrating the appropriateness of the design from the analysis, and assessment of the different variables involved in the preparation of PCs [47].

### Characterization of the optimal LUT-loaded PCs

#### Differential scanning calorimetry (DSC)

**Fig. 4:**
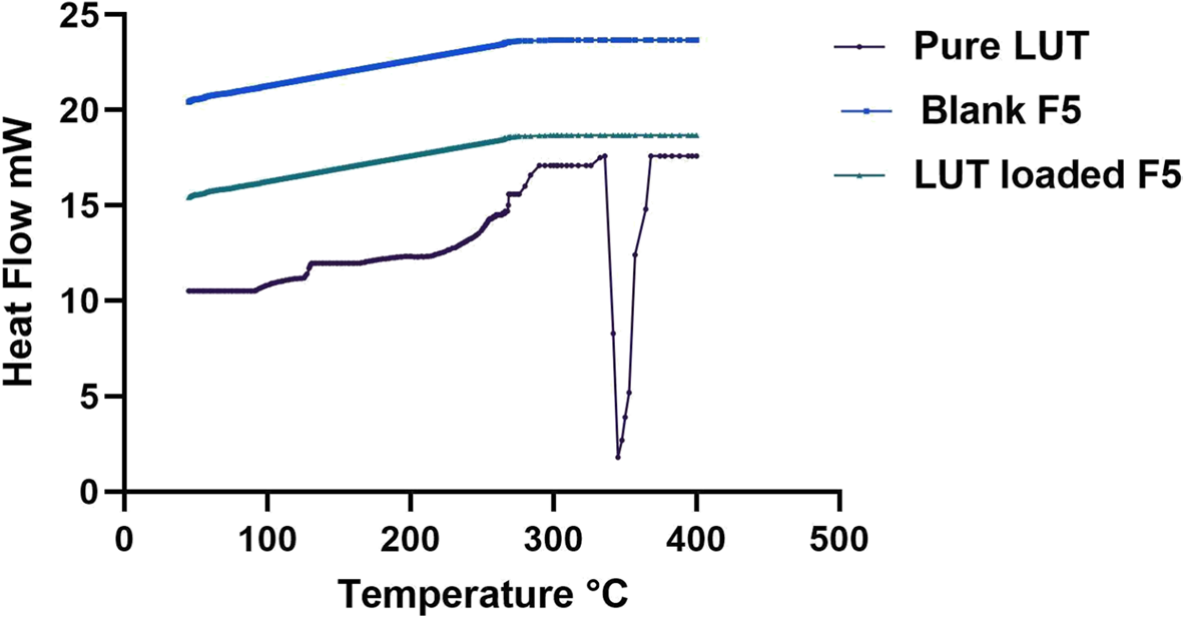
DSC thermograms of pure LUT, blank F5, and optimized LUT-loaded F5

DSC analysis was employed to determine the changes in the physical characteristics and level of crystallinity of LUT upon its loading in the optimized PCs. The different thermograms of pure LUT, plain optimized F5 formulation and the LUT-loaded F5 optimized formulation are shown in Fig. 4. The sharp, distinct peak corresponding to the melting point of LUT which is present at 344.7 ◦C in the thermogram of pure LUT is not present in the thermogram of the LUT-loaded F5 PC. This indicates the complete transformation of the drug from its crystalline state to an amorphous state due to its encapsulation within the prepared vesicles [34]. Moreover, neither the plain F5 nor the LUT-loaded F5 thermograms displayed any characteristic peak related to the excipients involved in the formulation of the PCs.

#### LUT in vitro release experiment

**Fig. 5:**
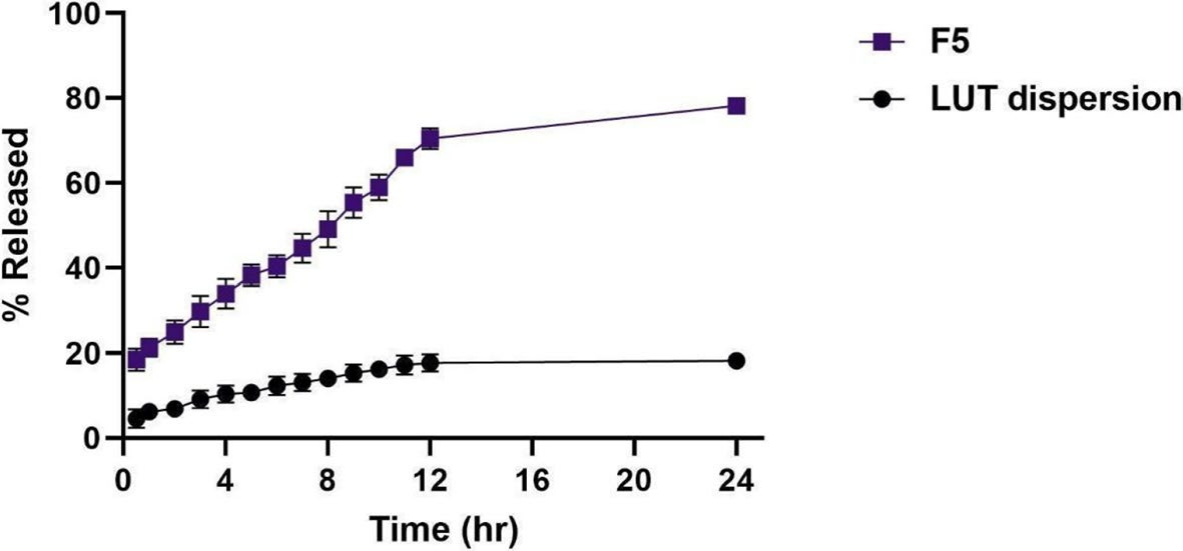
In vitro release profile of LUT from optimized LUT-loaded PCs (F5) compared to LUT dispersion

The in vitro release of LUT from F5 versus from the LUT dispersion was conducted to anticipate the effect of formulation on the solubilization and stability of the drug. The percentage of LUT released after 24 h (Q24h) reached a level of 78.1 ± 1.8%, compared to that of LUT suspension of 18.3 ± 2.1%. Figure 5 shows that the release of the drug from F5 occurs in two phases of the experiment: firstly, a rapid drug release phase in the first 2 h, followed by a delayed and more controlled phase over the next 24 h, due to the diffusion of the drug from the lipidic core to the outermost layers. This enhancement in the drug release rate from F5 compared to the LUT suspension may be due to the small PS of the prepared formula which in turn increases the surface area exposed to the dissolution medium, thus increasing the % of LUT released.

### Antibacterial activity against the tested MRSA strains: F5 formulation versus LUT dispersion

Several plant derived bioactive compounds have been proven to have antimicrobial activity [58]. Luteolin, a well-known plant secondary metabolite, has been demonstrated to show antibacterial activity against a wide range of bacteria such as *S. aureus, L. monocytogenes, E. coli,* and *Trueperella pyogenes* [59–61]. In this study, the optimized F5 formula and LUT dispersion were tested for their antibacterial activity against MRSA USA300 and four MRSA isolates using the broth microdilution method. A marked decrease in the MIC of the optimized F5 formulation with a value of 187.5 µg/mL (eightfold decrease) compared with that of the LUT dispersion (1500 µg/mL). Vancomycin was tested as the antibiotic positive control and showed MIC values ranging from 2–4 µg/mL with the tested strains. The optimized F5 formula had better antimicrobial activity which can be attributed to adhesion of the prepared vesicles to the bacterial cell membranes. Additionally, the PEGylation of the vesicles renders them more elastic and this may increase their accumulation and penetration ability to the bacterial cells. Thus, a higher percentage of the drug will be available at the bacterial cells, forming a thermodynamic activity gradient across the membranes which also improves the drug diffusion across the cellular membranes [50]. Clearly, the ceramide, phospholipids components and Brijs play a crucial role in improving LUT activity against MRSA infection. This is as a result of being able to induce alterations in the defense or barrier effects of the bacterial cell to the antibacterial activity and then helping LUT to diffuse of LUT through the bacterial cells.The minimum bactericidal concentrations (MBC) for both optimized F5 formulation and the LUT dispersion were assessed and revealed that they did not show any bactericidal activity. Elnady et al., also revealed the same findings, where naringenin loaded emulsomes were able to inhibit the growth of M. bovis five fold more than the pure naringenin [40]. Furthermore, our results can be confirmed with the results of Zakaria et al. who mentioned that the anti-MERS-CoV activity of resveratrol was greatly enhanced after being loaded in PEGylated emulsomes [48].

### Anti-virulence activity of the optimized F5 formulation

Methicillin-resistant *S. aureus* (MRSA) is known to possess numerous virulence factors and mechanisms, such as biofilm-forming ability, capsular polysaccharides, staphylococcal hemolysins, staphylococcal coagulase and staphyloxanthin [62]. As a result, the effect of the optimized F5 formulation in comparison with the LUT dispersion on some of these virulence factors was studied.

#### Optimized F5 formulation inhibited biofilm formation in the tested strains

**Fig. 6:**
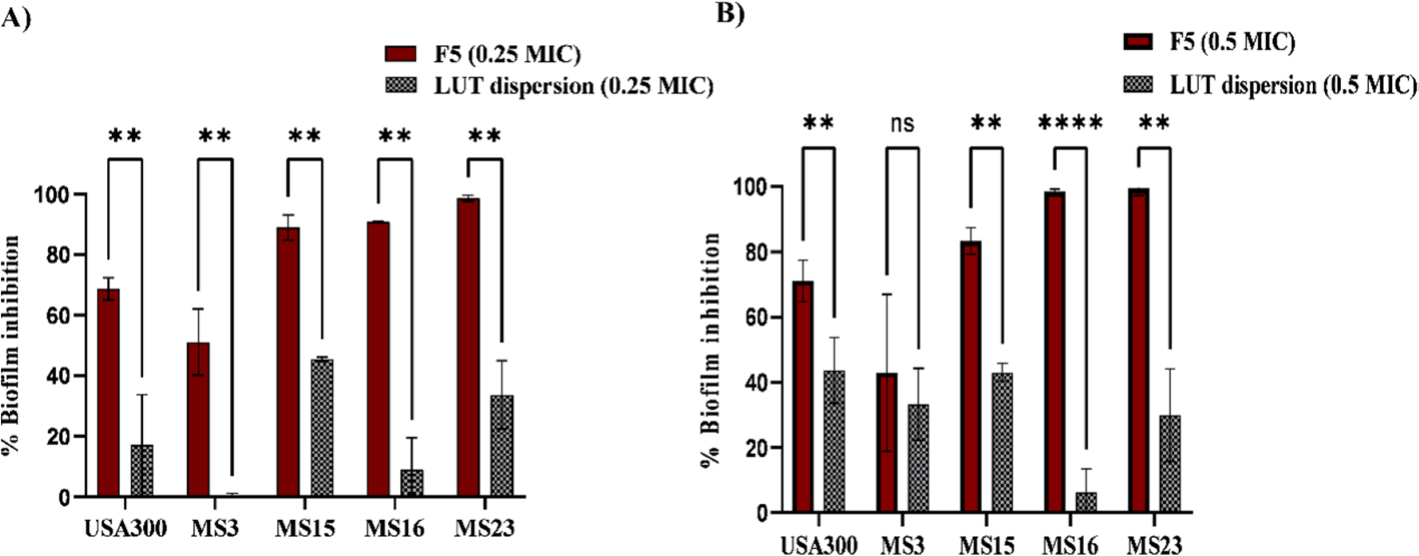
Percentage of biofilm formation inhibition with F5 compared with LUT dispersion at (**A**) 0.25 × and at (**B**) 0.5 × MIC against MRSA USA300 and four MRSA isolates. The data represents means of three independent experiments ± SD. Multiple unpaired t-test was used for statistical analysis. (**) indicates *p* ≤ 0.01 and (****) indicates *p* ≤ 0.0001. GraphPad prism (v9) was used for graph generation

Since Luteolin has been tested in previous studies for its antibiofilm activity against *S. aureus* [61, 63], the antibiofilm formation activity of F5 was compared to that of LUT dispersion. In the work reported herein, the antibiofilm formation activity of luteolin was enhanced by its incorporation in PCs by improving its permeability across bacterial membranes. The F5 formula used at either 0.25 × and 0.5 × MIC concentrations showed significant biofilm inhibition percentages in comparison to LUT dispersion with all of the tested strains (*p* value for 0.25 × MIC concentrations = 0.006, 0.008, 0.004, 0.002, 0.004, respectively) and (*p* value for 0.5 × MIC concentrations = 0.016, 0.56, 0.001, 0.00002, 0.002, respectively). The statistical analysis showed that only MS3 had non-significant inhibition in the concentration of 0.5 × MIC (Fig. 6).

#### Reduced pigmentation activity of MRSA strains with the F5 formula

Evaluation of the anti-pigment production activity of F5 on the selected MRSA strains was performed by the observation of staphyloxanthin pigment formation. Staphyloxanthin is a gold-colored carotenoid that is reported to be an important virulence factor in *S. aureus* [64]. It has been revealed that this pigment is involved in the evasion of *S. aureus* to a host’s immune system by being an antioxidant that helps in the detoxification of reactive oxygen species produced by the host immune system to combat the bacterium [65]. The golden yellow staphyloxanthin pigment is formed with MRSA strains, USA300, MS3, MS15, and MS23 but not with MS16. The results of F5 at both 0.25 × and 0.5 × MIC concentrations showed that it could inhibit the staphyloxanthin pigment of the four mentioned strains, indicated by turning the golden yellow color into colorless with these strains. These findings correspond to a previous study which investigated the role of different flavonoids including luteolin on the staphyloxanthin virulence factor in *S. aureus* [66]. We have also formulated luteolin in a previous study as an antimicrobial and anti-virulence drug [42] but in the current study F5 has the advantage of being more lipophilic due to the incorporation of ceramide thus allowing more of the drug to diffuse through the cells by increasing the ability of the formula to induce distortion in the bacterial cell membrane.

#### Optimized F5 formula suppressed α-hemolysin activity in the selected MRSA strains

**Fig. 7:**
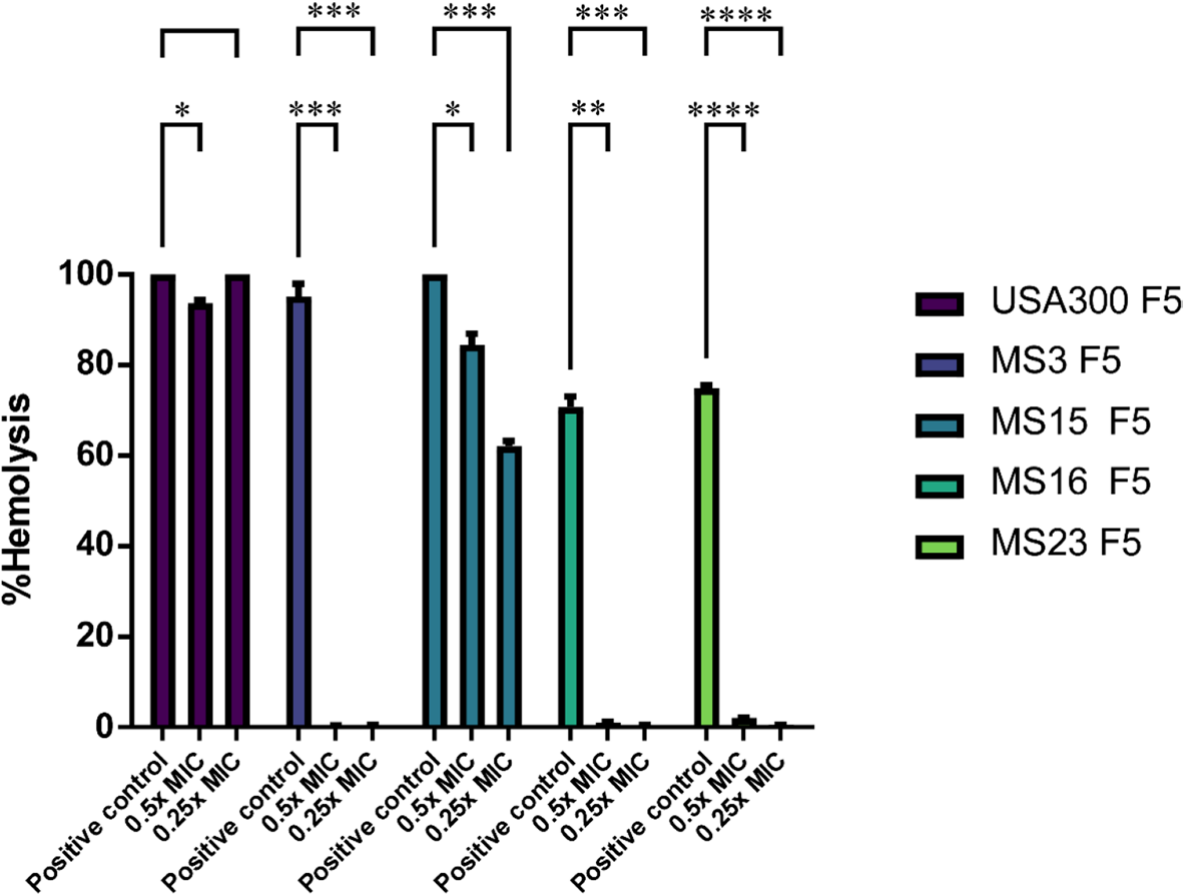
Anti–α-hemolysin activity of optimized F5 formula against MRSA strains. The figure shows the effect of F5 at its 0.25 × and 0.5 × MIC on α-hemolysin activity of MRSA strains represented by the decrease in % hemolysis of rabbit RBCs. The data represent means of three independent experiments of % hemolysis inhibition ± SD. Two-way ANOVA followed by Tukey’s multiple comparisons were used for statistical analysis. (*) indicates *p* ≤ 0.05, (**) indicates *p* ≤ 0.01, (***) indicates *p* ≤ 0.001 and (****) indicates *p* ≤ 0.0001. GraphPad prism (v9) was used for graph generation

The effect of F5 on α-hemolysin activity (Hla) was also assessed. Hla, a key virulence factor produced by *S. aureus*, causes damage and lysis in various cell types, including pneumocytes [67]. Therefore, it is a significant target for anti-virulence therapy aimed at reducing *S. aureus* pathogenesis within host cells. The results showed that F5 at 0.25 × and 0.5 × MIC could significantly inhibit the α-hemolysin activity of MRSA strains MS3, MS15, MS16, and MS23 as shown in Fig. 7 (*p* value for 0.25 × MIC concentrations = 0.5, 0.0005, 0.0006, 0.001, < 0.0001, respectively) and (*p* value for 0.5 × MIC concentrations = 0.02, 0.0005, 0.04, 0.001, < 0.0001, respectively). The α-hemolysin activity of USA300 was only significantly inhibited at 0.5 × MIC of F5. This result is consistent with that of the previous studies in that luteolin at sub-inhibitory concentration abolished the hemolysis activity of *S. aureus* [66, 68]. Other studies have explored the potential of different plant-based products as Hla inhibitors. He et al. (2021) [69] assessed the antimicrobial activity of four flavonoids, namely, baicalin, catechin, kaempferol, and quercetin, and showed that kaempferol and quercetin therapied the bacterial infection by inhibiting *S. aureus* α-hemolysin (Hla). Another study by Ping et al. (2018) [70] reported the effectiveness of prim-*O*-glucosylcimifugin (POG), a natural chromone compound, in inhibiting the secretion of Hla in *S. aureus* strain USA300 at sub-MIC concentrations.

### Assessment of potential disruption of F5 formula for the biofilm formation and cell wall structure of MRSA USA300

#### F5 could affect bacterial biofilm formed by MRSA USA300 strain

**Fig. 8:**
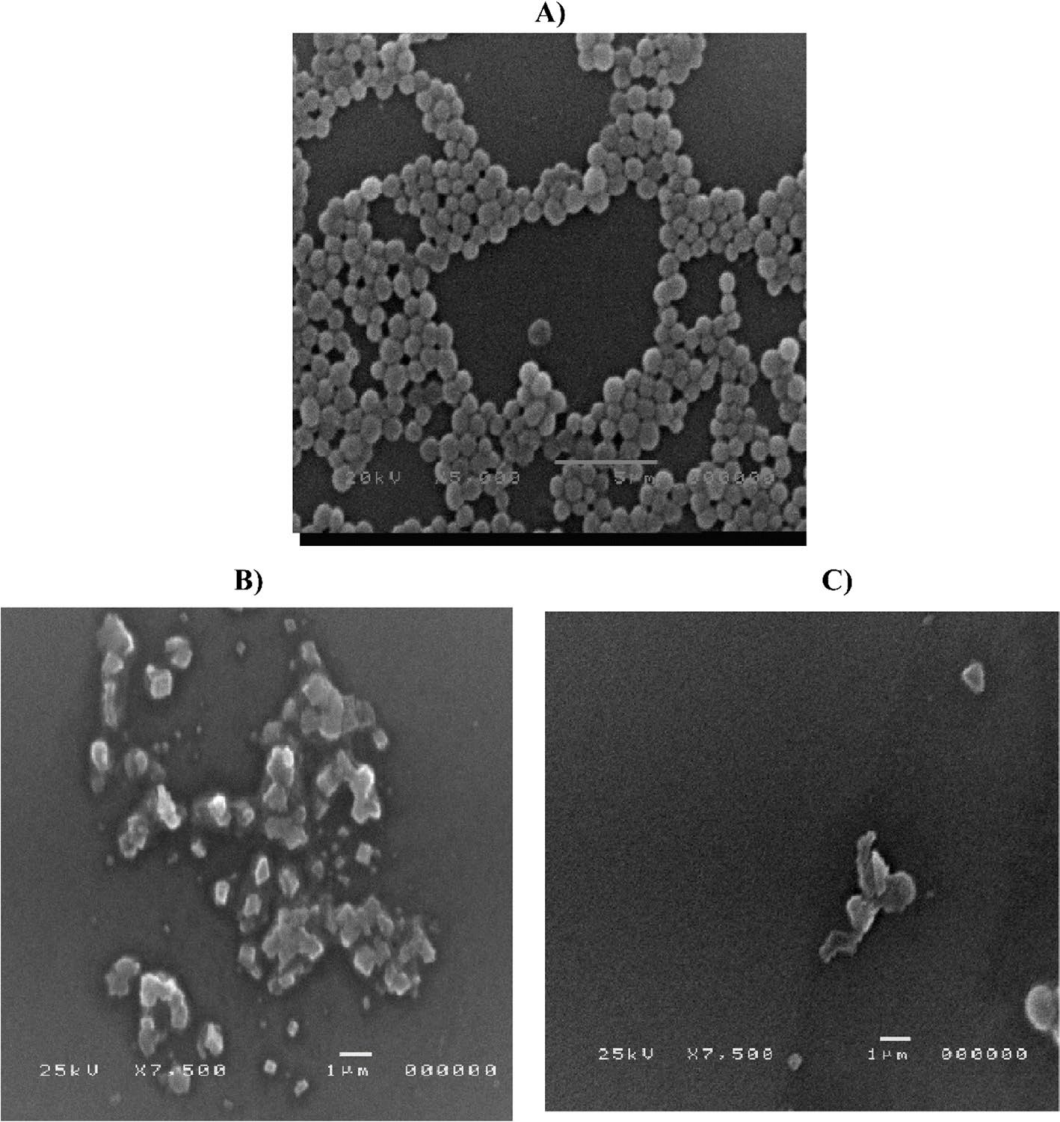
Photos of scanning electron microscope (SEM) of **A**) Control bacterial cells of MRSA USA300 showing intact bacterial aggregation and intact biofilm formation. **B** and** C** MRSA USA300 treated with sub-MIC of optimized F5 formula showing damage of formed biofilm and decrease in cellular adhesion. The resolution was 5.5 nm and magnification power of 15 × -200,000 × on a scanning electron microscope (JEOL model JSM-5200F, 25 kV, Tokyo, Japan)

Many natural products have demonstrated antibacterial properties through various mechanisms including disrupting cell wall and membrane integrity, suppressing protein expression and nucleic acid synthesis [71, 72]. This study aimed to understand the effect of luteolin on bacterial cells of MRSA. SEM was employed to explore the impact of F5 at its sub-MIC concentrations on the morphological features of MRSA USA300 biofilm. In the control of untreated bacterial cells, cellular adhesion and bacterial biofilm formation were observed (Fig. 8A). In contrast, F5 treatment led to biofilm disruption and reduced cellular adhesion and aggregation, highlighting its anti-biofilm activity (Figs. 8B and C). Targeting biofilm, a significant contributor to antibiotic resistance in MRSA infections, emphasizes the potential of F5 formula as a strategic therapeutic intervention against MRSA [73, 74].

#### F5 prompted cell wall damage in the bacterial cells of MRSA USA300 strain

**Fig. 9:**
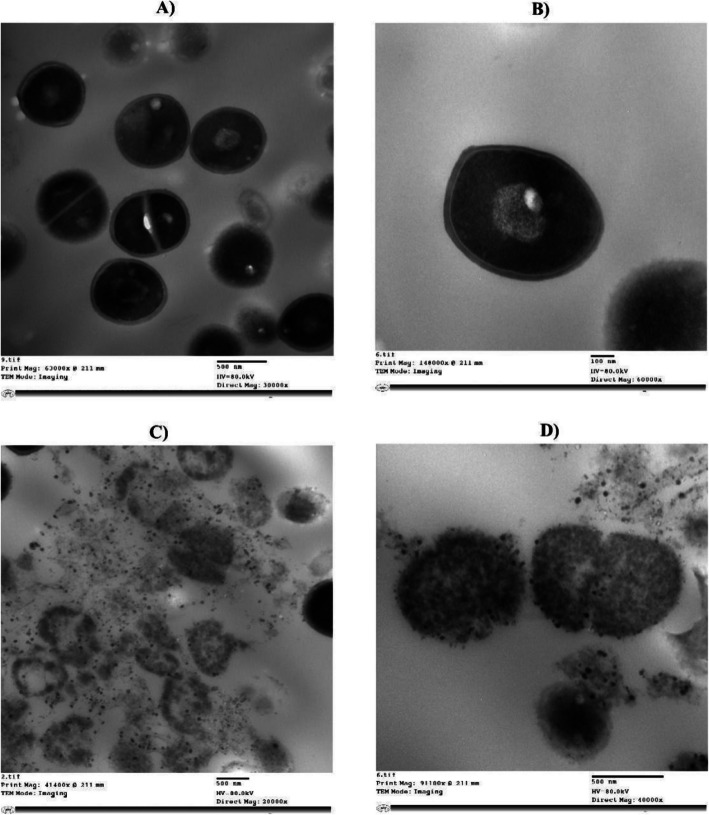
Photos of Transmission electron microscope (TEM) of: **A** and **B**) Control bacterial cells of MRSA USA300 showing intact bacterial cell wall and cellular content. **C** and **D** MRSA USA300 treated with MIC of optimized F5 formula showing cell wall and cell membrane damage and leakage of cellular content. The cells are noticed to be amorphous and vacuolated

The bacterial cell wall and membrane are vital for maintaining cell viability by preventing the leakage of intracellular components, which could impair bacterial growth [75–77]. TEM analysis of MRSA USA300 treated with the MIC of F5 revealed significant structural damage, including cell wall disruption, membrane integrity loss, and cytoplasmic leakage (Fig. 9). Thus, the cell wall and membrane of MRSA may be one of the targets of luteolin. This finding is consistent with previous studies, where LUT treatment was shown to damage *E. coli*, *S. aureus*, and *T. pyogenes* by disrupting the cell membrane, leading to cytoplasmic leakage [60, 78].

### In Vivo murine skin MRSA infection model

**Fig. 10:**
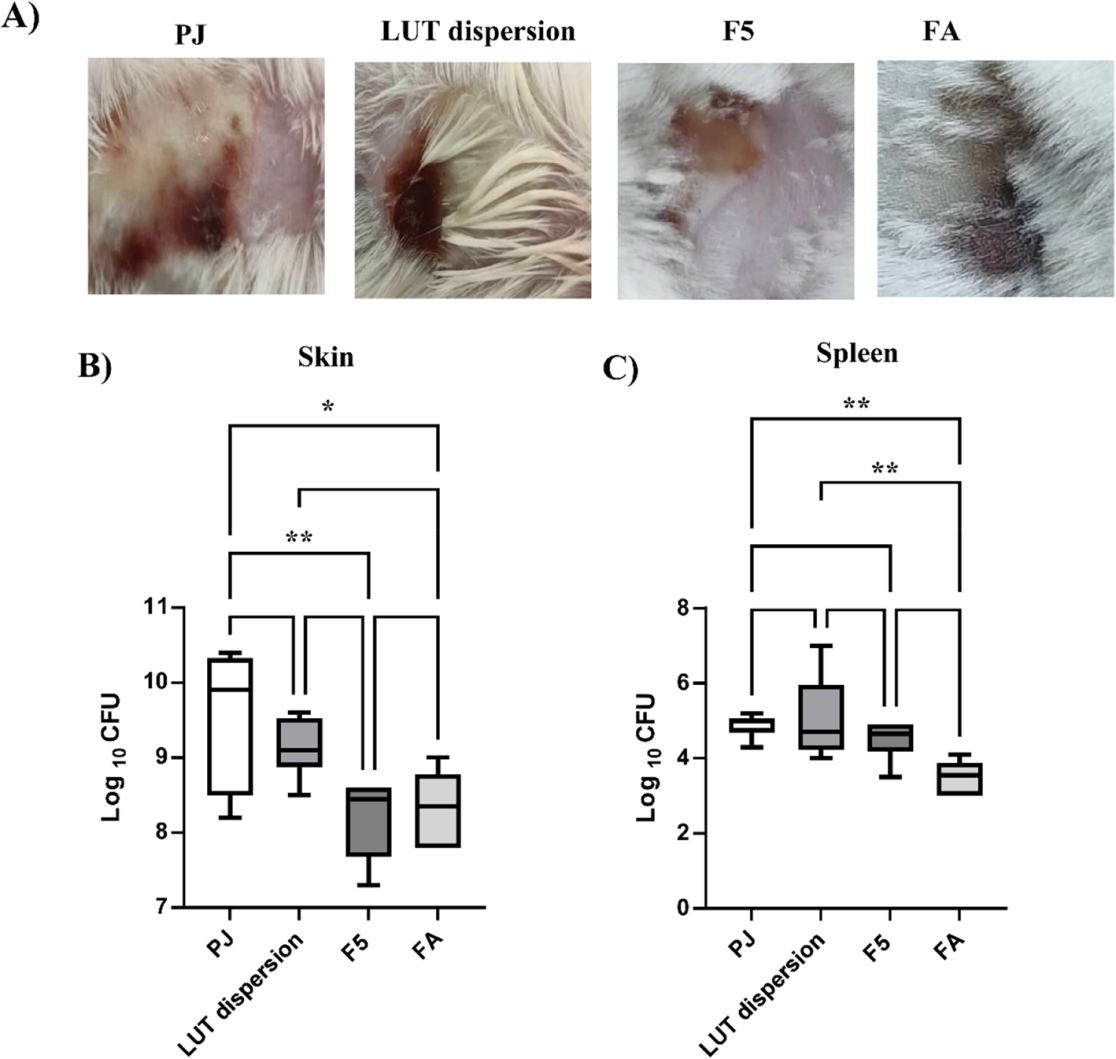
Optimized F5 formula effectively controls MRSA skin infections in a murine model. Photos of representative mice of each of the experimental groups on scarification day showing skin lesions (**A**). Box plots illustrate bacterial burden in skin lesions (**B**) and spleens (**C**) of the mice of the four groups. The data presented is the mean of the mice in each group ± SD. The whiskers span the difference between the minimum and maximum readings, the median was represented by the horizontal bars, and the mean of log10 CFU is indicated by ( +) sign. Statistical significance was tested by one-way ANOVA followed by Tukey’s Multiple Comparisons test, and *p* ≤ 0.05 was considered significant. (*) Means statistically significant difference exists between groups. The graph was generated by GraphPad Prism (v9)

F5 was tested in the Murine Skin MRSA Infection Model following its demonstrated potent in vitro antimicrobial activity. Skin lesions were observed 72 h post-subcutaneous infection with MRSA USA300. The infection site was then treated twice daily for four days, with petroleum jelly (PJ) as a negative control (Group 1), LUT dispersion in its 5 × MIC concentration (Group 2), F5 in its 5 × MIC concentration (Group 3), and 2% fusidic acid (FA) as a positive control (Group 4). The negative control group (PJ) maintained a persistent ulcerated skin lesion filled with pus and damage throughout the experiment, while the FA and F5 treated groups exhibited significant lesion recovery (Fig. 10A). This provided good evidence that the LUT dispersion and F5 were successful in containing the infection. Interestingly, the efficacy of the F5 formula was very comparable to that of the commercially available fusidic acid preparation as the bacterial counts of *S. aureus* recovered from both fusidic acid and F5 treated groups were significantly lower than those obtained in the PJ group (one-way ANOVA followed by Tukey’s multiple comparisons test, *p* = 0.0136 and 0.0059, respectively) (Fig. 10B). On the other hand, no significant difference was recorded between the negative control group and the group treated with LUT dispersion (Fig. 10B). As for controlling the dissemination of infection, bacterial counts in mouse spleens were quantified. Both the optimized formula and LUT dispersion decreased the bacterial count colonizing the spleen compared to that in the negative control group, however, no statistically significant difference was observed (Fig. 10C).

The in vivo mice model results confirmed the antimicrobial efficacy of both the LUT dispersion and F5 against *S. aureus*. Moreover, the results highlighted the superior antimicrobial activity of the F5 formulation, providing a significant advancement over the LUT dispersion. This provided good evidence that F5 was deemed to have enhanced efficacy compared to drug dispersion owing to its the ability to deliver higher amounts of the drug in solubilized form at the site of infection, induce distortion in barrier function of bacterial cell membrane, thus allowing more drug to diffuse throughout the bacterial cells. This improvement in the in vivo model came in agreement with Albash et al. who revealed that the activity of fenticonazole nitrate was greatly enhanced after being encapsulated in PCs [49]. Moreover, Elhabal et al. declared that cyclosporine-A (CsA) and dithranol loaded cerosomes could be proposed as a promising topical remedy for psoriasis as its reduced the pro-inflammatory cytokines and boost the skin penetration of both drugs compared to the unformulated ones [31].

Finally, the incorporation of ceramide was found to be very beneficial in remodulation of the integrity of skin layers and renewal of the natural barrier layer of the skin, so the overall skin condition will be enhanced subsequently.

## Conclusion

MRSA infections pose severe threats to global public health. Addressing this challenge demands innovative approaches. Thus, our study proposes a novel approach by developing luteolin loaded PEGylated cerosomes as nano-carriers to combat skin MRSA infections topically. The fabricated formulae with the aid of a 2^3^ full factorial design were characterized and then optimized for determination of an optimum formula (F5). F5 was shown to be significantly superior to a luteolin suspension in comparative in vitro and ex vivo studies. The comprehensive analysis of F5 demonstrated remarkable efficacy in inhibiting key virulence factors of MRSA, including biofilm formation, pigment production, and α-hemolysin activity. Moreover, SEM and TEM images revealed that the F5 formulation disrupts MRSA biofilm and damages bacterial cell walls. Interestingly, in vivo testing in a murine skin MRSA infection model validated the therapeutic potential of F5, with results comparable to the clinically used fusidic acid. Accordingly, F5 could be considered as a successful tool for management of MRSA skin infections.

## Data Availability

Data will be made available on request. To obtain access to the raw data analysed in your study, you can kindly contact one of the following: 1- Sally A. Mohamed, Microbiology and Immunology Department, Faculty of Pharmacy, Cairo University, Cairo, 11562, Egypt. sally.mohamed@pharma.cu.edu.eg 2- Walaa A. Eraqi, Microbiology and Immunology Department, Faculty of Pharmacy, Cairo University, Cairo, 11562, Egypt. walaa.eraqi@pharma.cu.edu.eg 3- Mohamed Y. Zakaria, Department of Pharmaceutics and Industrial Pharmacy, Faculty of Pharmacy, Port Said University, Port Said, 42526, Egypt; mohamed.zakaria@pharm.psu.edu.eg.
